# *Basella alba, Grewia asiatica, Solanum nigrum* and *Ficus carica* fruit extracts ameliorate the testicular histopathology induced by CCl_4_ exposure in albino mice: An experimental study

**DOI:** 10.18502/ijrm.v20i2.10502

**Published:** 2022-03-21

**Authors:** Syeda Nadia Ahmad, Khawaja Raees Ahmad, Usma Abdullah, Fiza Malik, Rabiyah Ali, Umara Amir-ud-din, Muhammad Ali Kanwal, Iram Inayat

**Affiliations:** ^1^Government Associate College for Women Bhera, District Sargodha, Pakistan.; ^2^Department of Zoology, University of Sargodha, District Sargodha, Pakistan.; ^3^Government Degree College for Women Mianwali, Sargodha, Pakistan.

**Keywords:** Basella alba, Grewia asiatica, Solanum nigrum, Ficus carica, testis.

## Abstract

**Background:**

Carbon tetrachloride (CCl ${}_{4}$ ) is extensively used in various industries and induces oxidative stress in humans. Unfortunately, it is one of the neglected potent male reproductive toxicants.

**Objective:**

The present research reports the testicular histopathology of CCl ${}_{4}$ and ameliorations by four medicinal fruit pulp extracts (FPEs) in mice.

**Materials and Methods:**

Sixty male albino mice were divided into six groups (10/group) as per the following: 1. Vehicle control (Vc); 2. CCl ${}_{4}$ (C); 3-6. CCl ${}_{4}$ +*Basella alba* (CBa), CCl ${}_{4}$ +*Solanum nigrum* (CSn), CCl ${}_{4}$ +*Ficus carica* (CFc) and CCl ${}_{4}$ +*Grewia asiatica* (CGa). Except for the control group, CCl ${}_{4}$ (0.1 mL of 0.2 mL kg ${}^{-1}$ ) was given to the animals in corn oil. The four plant extracts (0.1 mL each) were respectively given to the relevant FPE group animals for the next five consecutive days, while the animals in the Vc and CCl ${}_{4}$ groups received water instead of FPE.

**Results:**

The CCl ${}_{4}$ exposure led to various histometric and histological alterations (loss of interstitial tissue and various dislodged tailless spermatids with enlarged heads) that were recovered in all except *Solanum nigrum *FPE mice post-treatment. The micrometric data of testicular sections also indicated significant decline in the number of spermatogonia, while the cross-sectional area of the sperm heads remained significantly higher in the CSn and C groups.

**Conclusion:**

Except for *Solanum nigrum*,the three FPEs, especially *Ficus carica*,showed rehabilitative properties against CCl ${}_{4}$ exposure-related modifications in testicular histopathologies.

## 1. Introduction

CCl ${}_{4}$ is also called carbona, benzinoform and methane tetrachloride. Humans are exposed to CCl ${}_{4}$ through inhalation, dermal contact and ingestion. It is used in the production of chlorofluorocarbon refrigerants, foam-blowing agents, paints, plastics and as a solvent in metal cleaning and in fumigants (1). It damages body organs like the liver, kidney and testis (2).

Reactive oxygen species produced by CCl ${}_{4}$ exposure include trichloromethyl free radicals (CCl ${}_{3}$ ) and trichloroperoxyl radicals (CCl ${}_{3}$ O ${}_{2}$ ), which are highly reactive and increase lipid peroxidation (3). The radicals damage the membranes of testicular cells and lead to testicular toxicity. Biological exposure to CCl ${}_{4}$ due to its extensive environmental and occupational applications has led to various fertility issues like impaired spermatogenesis, hormonal changes, and degeneration of various differentiating spermatozoa (4). CCl ${}_{4}$ mainly targets the polyunsaturated fatty acids in the sperm membrane, causing lipid peroxidation and increased free radical concentration (5).

Antioxidant agents are chelating agents that scavenge free radicals and save cell membranes and eventually tissues from damage (6). The recent trends of research indicate the use of various medicinal fruit pulp extracts (FPEs) like turmeric, *Nepeta paulsenii, *ginger and cinnamon for the mitigation of noxious chemical agents like CCl ${}_{4}$ on the body organs, especially the testes (7-9). *Basella alba *contains certain distinct phytosterols like kaempferol and beta-sitosterol that are capable of reducing stress-related immuno-cellular responses (10). Likewise, *Grewia asiatica *and *Ficus carica *contain phytosterols, e.g campesterol and stigmasterol (11, 12), while *Solanum nigrum *contains various potent non-steroidal phytochemicals like solasonine, gallic acid and rutin (13).

In the present study, the bio-ameliorative potentials of the above-mentioned FPEs were compared against CCl ${}_{4}$ induced testicular histopathologies in adult albino mice. The novelty of this work was the use of commonly available wild medicinal plant extracts and the comparison of their ameliorative potential for testes. This is the first study to the best of our knowledge that has simultaneously addressed the testicular histopathologies of CCl ${}_{4}$ exposure and their mitigations by the use of the four medicinal FPEs of *Basella alba, Solanum nigrum, Ficus carica *and *Grewia asiatica.*

## 2. Materials and Methods

This experimental study was conducted for the exploration of the pathological effects of CCl ${}_{4}$ (a product of Riedal-de Haen, Germany, batch number 32215) and their ameliorations through various medicinal fruit extracts in albino laboratory mice.

### Animal conditions and dose groups

Sixty 7-8-wk-old, male Swiss Webster albino mice (*Mus musculus*) of 28-30 gr weight were kept in the animal house of the Department of Zoology, University of Sargodha under standard housing conditions. Animals were divided into six groups of 10 mice each.

a) Vehicle control (Vc) group: Received 0.1 mL corn oil (single dose) by gavage on day one, followed by free normal drinking water on days 2-6.

b) CCl ${}_{4}$ (C) group: Received single dose of 0.1 mL of 0.2 mL/kg CCl ${}_{4}$ solution in corn oil (containing 0.006 mL CCl ${}_{4}$ for the 30 gr animal weight) on day one by gavage, followed by free normal drinking water for days 2-6.

c) CCl ${}_{4}$ +*Basella alba *(CBa) group: Initial dose treatment was the same as in the C group on day one, followed by 0.1 mL freshly obtained FPE of *Basella alba* for days 2-6 every 12 hr.

d) CCl ${}_{4}$ +*Solanum nigrum *(CSn) group: Initial dose treatment was the same as in the C group on day one, followed by 0.1 mL freshly obtained FPE of *Solanum nigrum* for days 2-6 every 12 hr.

e) CCl ${}_{4}$ +*Ficus carica* (CFc) group: Initial dose treatment was the same as in the C group on day one, followed by 0.1 mL freshly obtained FPE of *Ficus carica* for days 2-6 every 12 hr.

f) CCl ${}_{4}$ +*Grewia asiatica *(CGa) group: Initial dose treatment was the same as in the C group on day one, followed by 0.1 mL freshly obtained FPE of *Grewia asiatica* for days 2-6 every 12 hr.

### Fruit extract preparation

Fresh fruits of *Grewia asiatica *and *Ficus carica* were obtained from the market whereas the *Basella alba *and *Solanum nigrum* fruits were collected from the suburbs of Sargodha city. All fruits were thoroughly washed, air dried, and crushed gently. Finally, the FPEs of all mentioned plants were prepared by following the lab protocol (14–17). The freshly thawed FPEs were provided to the animals of the relevant groups.

### Preparation of CCl ${}_{4}$ solution

The laboratory grade CCl ${}_{4}$ was dissolved in corn oil (15:85 v/v respectively) to prepare the stock solution. This stock CCl ${}_{4}$ solution was further diluted (6:94 v/v) to prepare the required strength of 0.006 mL CCl ${}_{4}$ /0.1 mL (single dose volume for each animal in the relevant experimental groups).

### Animal dissections, organ recovery and histological studies

Animals were sacrificed through cervical dislocation on day seven. Testes were recovered and their general appearance and weight were recorded. One testis from each animal in all the groups was randomly selected for smear preparation while the other was processed for microtomy and hematoxylin and eosin staining. The smears and serial sections were finally observed for various landmarks of toxicological and ameliorative activities.

### Digital histometry of testis and testicular smear

Digital histometry of testicular sections was conducted in CorelDRAW 11 under calibrated scales (cm: µ). Digital images (x400 magnification) of randomly selected sections of testes and testicular smears from each group were used for this purpose. The mean measurements of spermatogonia along the basement membrane µ ${}^{-1}$ , mean cross-sectional area (CSA) of the sperm head, mean tail length of sperm and mean number of whirls of spermatogonia/seminiferous tubules were estimated. The CSA of the sperm head was calculated using the formula CSA = (length × width)/4*π.

### Ethical considerations

All procedures of this study were approved by the ethical committee of the Department of Zoology, University of Sargodha, Sargodha, Pakistan (Code: SU/Zol/2665).

### Statistical analysis

For the histometric and micrometric analyses, mean ± standard error of the mean and mean CSA were calculated. The data were further analyzed using the Statistical Package for the Social Sciences (SPSS.13) software, a brand of International Business Machines Corporation (IBM), SPSS Statistics, New York, USA. Two-way ANOVA followed by Tukey's Multiple Range Test were used. The mean values at p < 0.05 were considered as significant.

## 3. Results

### Histological results of testes

**Figure 1:**
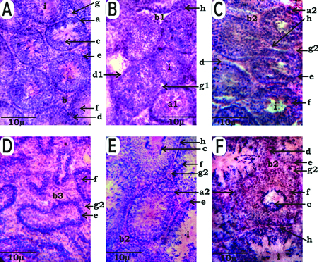
Histological sections of testes (x400 magnification, Hematoxylin and eosin-stained sections). A: Vc, B: C, C: CBa, D: CSn, E: CGa, F: CFc, a: Normal seminiferous tubules, a1: Damaged seminiferous tubules, a2: Regenerated seminiferous tubules, b: Normal interstitial tissue, b1: Degenerated interstitial tissue, b2: Regenerated interstitial tissue, b3: Fibrosis of interstitial tissue, c: Normal sperm distribution pattern, d: Basement membrane, d1: Damaged basement membrane, e: Spermatogonia, f: Spermatocytes, g: Leydig cells, g1: Degenerated Leydig cells, g2: Regenerated Leydig cells, h: Pericytes, i: Lumen in seminiferous tubules.

The histological results of the testes showed that in the Vc group, rounded seminiferous tubules delimitated externally with the basement membrane were well visualized. In each tubular section, the spermatogonia, primary spermatocytes, spermatids and spermatozoa were found arranged concentrically peripheral to central whirls. Healthy and normal interstitial tissue was found filling the spaces among the tubular sections (Figure 1A). Contrary to these, the C group sections showed irregularly shaped seminiferous tubules. The internal arrangement of the spermatogenic cells was also disrupted. These cells were found rather scattered and poorly differentiated. Clumps of spermatogonial cells at peripheral margins of the tubular sections were also seen (Figure 1B). In the CBa slides, rehabilitative ultrastructural architectural arrangements were observed as shown by the partial rehabilitation of whirls of spermatogenic cells - an indication of the restoration of spermatogenesis. Healthy interstitial tissue was also observed with many regenerated Leydig cells (Figure 1C). In the CSn group slides, the size of the seminiferous tubules was much reduced as compared to the Vc group. The shape of the seminiferous tubules was also variable showing polygonal irregular margins. The spermatogonia, primary spermatocytes, secondary spermatocytes and spermatids were not present in proper alignment. This situation seemed more deteriorated than the arrangement of spermatogonia and spermatocytes in the C group. Moreover, all seminiferous tubules were devoid of differentiating spermatids and spermatozoa. The interstitial tissue showed necrosis of the Leydig cells (Figure 1D). The obvious ameliorative and regenerative indications were observed in the CGa group such as that the CSA of the seminiferous tubular sections were much enlarged. The lumen of the seminiferous tubules was occupied with an enormous number of mature spermatozoa. Regeneration of interstitial tissue was observed from the pericytes resulting in the rehabilitation of the Leydig cells (Figure 1E). The best regenerative signs were seen in the CFc group, including rounded and much enlarged seminiferous tubular sections as compared to the C group. However, small aggregates of spermatozoa were present in the central luminal spaces of the seminiferous tubules. The interstitial tissues seemed more rehabilitated than those of the C group (Figure 1F).

### Mean weight parameters for animals and organs (testes)

The mean group body weights in all five experimental groups were significantly lower than the Vc group on day seven; however, the maximum decrease was recorded in the CSn and C groups. The mean testicular weight was also significantly lower in the C and CSn groups than in the rest of the four groups (Table I).

### Histometric results of testes

The micrometric data of the testicular sections also indicated a significantly lower (p < 0.001) mean number of spermatogonia along the length (1 µ) of the basement membrane in the C group, compared with the rest of the five groups. The mean CSA of the sperm heads was significantly higher in the CSn and C groups than in the other four groups, and the mean tail length of the sperm was significantly lower in the C and CSn groups than in the rest of the four groups. Likewise, the mean number of whirls of spermatogonia per seminiferous tubule was significantly lower in the C group than in the other five groups (Table II).

### Testicular smear results

**Figure 2:**
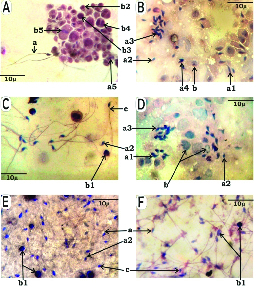
Testicular smears (x400 magnification, Hematoxylin and eosin-stained sections). A: Vc, B: C, C: CBa, D: CSn, E: CGa, F: CFc, a: Normal sperms with parakeet beak-shaped head, small neck, middle piece and well developed straight and long tail, a1: Tailless sperm spermatids with crow beak-shaped head, a2: Micronuclei spermatid head, a3: Cluster of spermatids with probably halted spermiogenesis, a4: Two headed spermatid, a5: Normal spermatids, b: Necrotizing spermatogenic cells (Spermatogonia and spermatocytes), b1: Healthy spermatogenic cells (Spermatogonia and spermatocytes), b2: Normal spermatogonia, b3: Primary spermatocyte, b4: Secondary spermatocyte, b5: Mitotic spindle of primary spermatocyte, c: Still sperms with normal tail.

The testicular smears in the Vc group showed a large number of spermatozoa with normal parakeet beak-shaped heads and a long straight tail. Some spermatogenic cells and partially formed spermatozoa at various stages of spermiogenesis were also seen along with hormone-secreting interstitial Leydig cells (Figure 2A). In the CCl ${}_{4}$ group, maturing and mature spermatozoa were rarely observed; however, various spermatids with a small rudimentary tail or no tail were frequently observed. Clumps of interstitial cells were rarely seen (Figure 2B). In the CBa group slides, many maturing and mature spermatozoa, spermatocytes, spermatogonia, spermatids and some aggregations of interstitial cells were observed. Nevertheless, many of these spermatogenic and interstitial cells were also found showing intracellular vacuolations, indicating signs of intracellular toxicity and necrosis (Figure 2C). In the CSn group slides, the situation seemed to be worse in terms of a large number of necrotizing spermatids and other spermatogenic cells with no matured or maturing spermatozoa (Figure 2D). In the CGa group slides, spermatids were frequently observed. Some maturing spermatozoa were also found while the other spermatogenic cells like spermatogonia and spermatocytes were also observed. The frequency of occurrence of spermatogonia seemed even better than in the CBa group (Figure 2E). In the CFc group, although all spermatogenic cells (spermatogonia, spermatocytes, maturing spermatids and maturing and matured spermatozoa) and a few clumps of interstitial cells were present, some spermatogenic cells also showed signs of apoptosis (cytoplasmic vacuolations) (Figure 2F).

**Table 1:** Mean animal weight comparison among different groups on day zero and day seven of the study


**Weight parameters**	**Groups**						**p-value**
	**V ${}_{𝐂}$ **	**C**	**CSn**	**CBa**	**CFc**	**CGa**	
**Mean animal weight** ** at day one (gr)**	30.52 ± 0.22 ${}^{a}$	30.35 ± 0.14 ${}^{a}$	30.15 ± 0.11 ${}^{a}$	30.03 ± 0.02 ${}^{a}$	30.25 ± 0.26 ${}^{a}$	30.20 ± 0.08 ${}^{a}$	≤ 0.05
**Mean animal weight** ** at day seven (gr)**	30.16 ± 0.67 ${}^{a}$	27.08 ± 0.64 ${}^{b}$	26.02 ± 0.64 ${}^{c}$	28.32 ± 0.64 ${}^{e}$	29.05 ± 0.66 ${}^{d}$	29.60 ± 0.64 ${}^{d}$	≤ 0.001
**Mean testes weight** ** at day seven (gr)**	0.13 ± 0.009 ${}^{a}$	0.08 ± 0.005 ${}^{b}$	0.056 ± 0.008 ${}^{c}$	0.11 ± 0.002 ${}^{a}$	0.12 ± 0.004 ${}^{a}$	0.12 ± 0.002 ${}^{a}$	≤ 0.001
Data shown as Mean ± SEM, Statistical analysis was by two-way ANOVA. ${}^{a,\;b,\;c,\;d,\;e}$ : Any two groups not sharing a lowercase letter differed significantly from each other (post hoc analysis). V_C_: Vehicle control, C: CCl ${}_{4}$ , CSn: CCl ${}_{4}$ +*Solanum nigrum*, CBa: CCl ${}_{4}$ +*Basella alba*, CFc: CCl ${}_{4}$ +*Ficus carica*, CGa: CCl ${}_{4}$ +*Grewia asiatica*							

**Table 2:** Micrometry (CSA) of the various dimensions of the spermatozoa and number of spermatogonia along basement membrane of 1 µ


**Micrometric** ** parameters**	**Groups**						**p-value**
	**V ${}_{𝐂}$ **	**C**	**CSn**	**CBa**	**CFc**	**CGa**	
**Mean number of ** ** spermatogonia** ** along basement** ** membrane of 1 µ **	1.53 ± 0.16 ${}^{a}$	1.14 ± 0.14 ${}^{b}$	1.71 ± 0.15 ${}^{c}$	1.29 ± 0.11 ${}^{d}$	1.79 ± 0.16 ${}^{e}$	1.30 ± 0.12 ${}^{d}$	< 0.001
**Mean CSA of sperm** ** head (µm²)**	6.28 ± 0.29 ${}^{a}$	11.92 ± 0.47 ${}^{b}$	12.13 ± 1.06 ${}^{b}$	6.66 ± 0.41 ${}^{a}$	6.99 ± 0.27 ${}^{a}$	6.06 ± 0.19 ${}^{a}$	< 0.001
**Mean tail length of ** ** sperm (µm²)**	52.70 ± 2.07 ${}^{a}$	6.61 ± 0.44 ${}^{b}$	5.04 ± 0.86 ${}^{b}$	56.67 ± 0.89 ${}^{e}$	48.99 ± 0.93 ${}^{c}$	32.82 ± 1.64 ${}^{d}$	< 0.001
**Mean number of** ** spermatogonial** ** whirls/seminiferous** ** tubules**	2.56 ± 0.07 ${}^{a}$	0.91 ± 0.08 ${}^{b}$	2.38 ± 0.07 ${}^{a}$	1.32 ± 0.06 ${}^{e}$	3.12 ± 0.09 ${}^{d}$	1.96 ± 0.09 ${}^{c}$	< 0.001
Data shown as Mean ± SEM, Statistical analysis was by two-way ANOVA. ${}^{a,\;b,\;c,\;d,\;e}$ : Any two groups not sharing a lowercase letter differed significantly from each other (post hoc analysis). V_C_: Vehicle control, C: CCl ${}_{4}$ , CSn: CCl ${}_{4}$ +*Solanum nigrum*, CBa: CCl ${}_{4}$ +*Basella alba*, CFc: CCl ${}_{4}$ +*Ficus carica*, CGa: CCl ${}_{4}$ +*Grewia asiatica*							

## 4. Discussion

In contrast to testicular sections in the Vc group (Figure 1A) the CCl ${}_{4}$ group showed severely damaged interstitial tissue and delocalized spermatogenic cells. The interstitial tissue showed severe damage as reflected by the cellular obliterations leaving wide empty spaces containing small masses of the androgenic hormone secreting Leydig cells (Figure 1B). These histoarchitectural damages were logical consequences of lipid peroxidation in the cell membrane and the membranous organelles leading to cellular necrosis in the actively metabolizing interstitial and Sertoli cells (Figure 2B).

The most appreciable rehabilitation from testicular pathologies was seen in the CBa group, followed by the CGa and CFc groups (Figures 1C, 1E and 1F, respectively). However, in the CSn group, worse conditions were observed with extreme shrinkage of the tubular sections of the CCl ${}_{4}$ exposure damage, and no signs of recovery (Figure 1D). The prominent signs of rehabilitation of steroidogenesis and spermatogenesis in the Cba, CGa and CFc groups were the at least partially regenerated interstitial tissue and the realigned sperm-producing cells within the tubules. This included re-arrangement of whirls of spermatogonia along the basement membrane, followed by the inner whirls of spermatogenic cells at various stages of spermiogenesis (Figures 1C, 1E and 1F, respectively). These rehabilitative activities were likely partially attributable to the unique blends of phytosterols like sitosterol, stigmasterol and campesterol found in the FPEs of *G. asiatica*, *B. alba *and *F. carica* (10–13) that mimic the role of male sex hormones and thus logically contribute towards stress alleviation, by promoting the anabolic activities in the testis, and thereby assisting the restoration and rehabilitation of spermatogenesis. The process of testicular histoarchitectural rehabilitation may involve the activation of dormant pericytes present at the margins of the seminiferous tubules by the androgenic phytosterols present in these FPEs. The elicited pericytes may rehabilitate the functional Leydig cells through proliferation (18). Also, mitotic reactivation of the scanty spermatogonial cells that escaped CCl ${}_{4}$ toxicity may have been involved in the restoration of spermatogenesis (19). This idea is further supported as significantly fewer spermatogonia were found per µ along the basement membrane in the C group than in that of the Vc, CBa, CFc and CGa groups. The best rehabilitative effect on the number of spermatogonia was observed in the CFc group, which perhaps could be ascribed to the large battery of unique phytosterols (12).

The increased number of spermatogonia in the CSn group was probably partly due to the extreme shrinkage of the seminiferous tubules observed because of the anti-steroidogenic effects of the solasonine in *S. nigrum* (Table II), which may have halted the surviving spermatogonia and their role in the process of spermatogenesis (20). The histometric parameters of the developing sperms indicated gross alterations in the head and tail sizes in the CCl ${}_{4}$ and CSn groups (Table II) that must have been a consequence of halted spermiogenesis owing to severe damage to the Sertoli cells after CCl ${}_{4}$ exposure; this damage may have been worse in the CSn group due to its solasonine (21).

**Figure 3:**
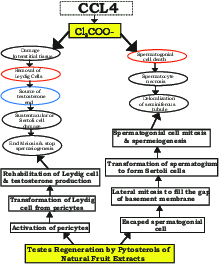
Flow chart showing the oxidative stress created by CCl ${}_{4}$ and the role of plant extracts as antioxidants during testes rehabilitation.

Rehabilitation of the spermatic tissue involved differentiation of primary spermatogonial cells and partial rehabilitation of the stressed Sertoli cells in the plant extracts of the CGa, CBa and CFc groups. In the CCl ${}_{4}$ treated group, cellular damage was indicated by frequently observed loss of maturing spermatozoa and necrotizing spermatogenic cells. This was a logical consequence of an acute nutritional deficiency in the spermatogenic cells because of their general dislodgement from the narcotized nurturing Sertoli cells on account of direct CCl ${}_{4}$ exposure (22). Nevertheless, the direct CCl ${}_{4}$ exposure might also have contributed to the oxidative damage to the plasma membrane of the spermatogenic cells and maturing spermatozoa (5). Additionally, this exposure may have also interfered with the process of mitosis and meiosis in the spermatogenic cells (23, 24). The further negative consequences observed in the CSn group indicated that the *S. nigrum* FPE further enhanced CCl ${}_{4}$ toxicity, which may have been attributable to solasonine selectively damaging the process of steroidogenesis (25, 26). Among the other three groups treated with the FPEs, the *F. carica* group showed the best rehabilitation of spermatogonial cell populations which can likely be attributed to its phytosterols. Thus, the *F. carica *treatment might have led to the fastest alleviation of oxidative stress, helping in rapid rehabilitation of the interstitial tissue and further resulting in natural androgen secretion, ultimately causing almost complete rehabilitation of the processes of meiosis and spermiogenesis (Figure 3).

## 5. Conclusion

Out of the four plant extracts used in this study, unlike *Solanum nigrum*, *Ficus carica*, *Grewia asiatica* and *Basella alba* showed good capacity for testicular histoarchitectural rehabilitation after CCl ${}_{4}$ exposure, an indication of the importance of phytosterols and natural antioxidants. This study demonstrated the importance of ethnomedicinal compounds against the testicular toxicological damage of CCl ${}_{4}$ , an environmental chemical with multiple occupational exposure risks. The findings indicated a need for further investigation into the antioxidant ingredients of these plants and their biochemical approach.

##  Conflict of Interest

The authors declare that there is no conflict of interest.

## References

[B1] Sherry D, McCulloch A, Liang Q, Reimann S, Newman PA (2018). Current sources of carbon tetrachloride (CCl4) in our atmosphere. Environ Res Lett.

[B2] Jayakumar T, Sakhtivel M, Thomas PA, Geraldine P (2008). Pleurotus ostreatus, an oyster mushroom, decreases the oxidative stress induced by carbon tetrachloride in rat kidneys, heart and brain. Chem Biol Interact.

[B3] Khan S, Rahman M, Kabir F, Nahar K, Mamun F, Lasker Sh, et al (2020). Trichosanthes dioica Roxb prevents hepatic inflammation and fibrosis in CCl4-induced ovariectomized rats. Clin Nutr Exp.

[B4] Elsawy H, Badr GM, Sedky A, Abdallah BM, Alzahrani AM, Abdel-Moneim AM (2019). Rutin ameliorates carbon tetrachloride (CCl4) induced hepatorenal toxicity and hypogonadism in male rats. Peer J.

[B5] Foaud MA, Kamel AH, Abd El-Monem DD (2018). The protective effect of N-acetyl cysteine against carbon tetrachloride toxicity in rats. J Basic Appl Zool.

[B6] Yashin A, Yashin Y, Xia X, Nemzer B (2017). Antioxidant activity of spices and their impact on human health: A review. Antioxidants.

[B7] Eric EU, Adolphus E (2020). Ameliorative effects of turmeric extract against CCl4 induced liver and kidney injury in adult Wistar rat. Asian J Immunol.

[B8] Samad A, Ijaz MU, Ashraf A, Sajid M, Imran M, Abbas K, et al (2020). Methanolic extract of Nepeta paulsenii as an ameliorative agent against CCl4 induced testicular damage in male albino rats. J King Saud Univ Sci.

[B9] Mazani M, Ojarudi M, Banaei Sh, Salimnejad R, Latifi M, Azizi H, et al (2020). The protective effect of cinnamon and ginger hydro‐alcoholic extract on carbon tetrachloride‐induced testicular damage in rats. Andrologia.

[B10] Singh A, Dubey PK, Chaurasiya R, Mathur N, Kumar G, Bharati S, et al (2018). Indian spinach: An underutilized perennial leafy vegetable for nutritional security in developing world. Energ Ecol Environ.

[B11] Khan RS, Asghar W, Khalid N, Nazir W, Farooq M, Ahmed I, et al (2019). Phalsa (Grewia asiatica L) fruit berry a promising functional food ingredient: A comprehensive review. J Berry Res.

[B12] Gani G, Fatima T, Qadri T, Beenish Jan N, Bashir O (2018). Phytochemistry and pharmacological activities of fig (Ficuscarica): A review. Inter J Res Pharm Pharmaceut Sci.

[B13] Rani YS, Reddy VJ, Basha ShJ, Koshma M, Hanumanthu G, Swaroopa P (2018). A review on Solanum nigrum. World J Pharm Pharmaceut Sci.

[B14] Ahmad KR, Sial B, Amiruddin U, Bilal MA, Raees K, Abbas T, et al (2016). Hepato-curative and regenerative potentials of wild olive (Oleaferruginea) fruit pulp extracts against fluoride-induced toxicity in mice: A histopathological study. Fluoride.

[B15] Satheesh NK, Gurushanthaiah M, Kavimani M, Prabhu K, Lokanadham S (2017). Hepatic regeneration by Baila alba fruit extract against chromium (VI) induced toxicity. Int J Adv Biotech Res.

[B16] Inayat I, Ahmad SN, Batool H, Younis A, Abbas T, Batool S, et al (2019). Mitigations of Syzygium cummini fruit extract on CdCl2 induced histopathological changes in laboratory mouse epididymis. Egypt Acad J Biol Sci.

[B17] Inayat I, Ahmad SN, Muqaddas I, Suleman S, Siddique S, Batool AI, et al (2020). Hepato-protective capacity of Morus (Morusmacroura) fruit extract against lead treated mice: A histopathological study. Int J Biosci.

[B18] Ye L, Li X, Li L, Chen H, Ge RS (2017). Insights into the development of the adult Leydig cell lineage from stem Leydig cells. Front Physiol.

[B19] Lee KH, Lee WY, Kim DH, Lee SH, Do JT, Park Ch, et al (2016). Vitrified canine testicular cells allow the formation of spermatogonial stem cells and seminiferous tubules following their xenotransplantation into nude mice. Sci Rep.

[B20] Wang Y, Xiang L, Yi X, He X (2017). Potential anti-inflammatory steroidal saponins from the berries of Solanum nigrum L (European black nightshade). J Agric Food Chem.

[B21] Jolene EY, Michael JB (2010). Dominican medicinal plants: A guide for health care providers. 2nd Ed New York: The New York Botanical Garden;.

[B22] Bubnov RV, Drahulian MV, Buchek PV, Gulko TP (2018). High regenerative capacity of the liver and irreversible injury of male reproductive system in carbon tetrachloride-induced liver fibrosis rat model. EPMA J.

[B23] Ray S, Murmu N, Adhikari J, Bhattacharyya S, Adhikari S, Banerjee S (2014). Inhibition of Hep G2 hepatic cancer cell growth and CCl₄ induced liver cytotoxicity in swiss albino mice by mahua extract. J Environ Pathol Toxicol Oncol.

[B24] Wang TY, Li Y, Wu X, Yang XL, Wang Y, Wang D (2018). Protective effects of melatonin on CCl4 induced acute liver damage and testicular toxicity in rats. Indian J Pharm Sci.

[B25] Edmonds JM, Chweya JA, Heller J, Engels JMM (1997). Black nightshades Solanum nigrum L. and related species Rome, Italy: IPK and IPGRI Publisher;.

[B26] Ahmed R (2019). Steroidal glycoalkaloids from Solanum nigrum target cytoskeletal proteins: An in silico analysis. Peer J.

